# Supplementary material for: A Variant-Centric Analysis of Allele Sharing in Dogs and Wolves
Source: Genes (Basel). 2024 Sep 5;15(9):1168. doi: 10.3390/genes15091168 (PMC11431226; doi:10.3390/genes15091168)
Supplement: Supplementary file 1 [file genes-15-01168-s001.zip › genes-3173531-supplementary.pdf]

A variant centric analysis of allele sharing in dogs and wolves

Matthew W. Funk[1] and Jeffrey M. Kidd[1,2*]

[1]Department of Computational Medicine & Bioinformatics, University of Michigan, Ann Arbor, MI
[1]Department of Human Genetics, University of Michigan, Ann Arbor, MI

Supplementary Tables and Figures

Supplementary Table 1. Village Dog Population Assignments

East Asia				Central Asia	Africa
VILLCN000001	VILLCN000002	VILLCN000003	VILLCN000004	VILLAZ000001	VILLCG000001
VILLCN000005	VILLCN000006	VILLCN000007	VILLCN000008	VILLAZ000002	VILLCG000002
VILLCN000009	VILLCN000011	VILLCN000012	VILLCN000013	VILLAZ000003	VILLCG000003
VILLCN000014	VILLCN000015	VILCN000016	VILLCN000018	VILLAZ000004	VILLCG000004
VILLCN000019	VILLCN000020	VILLCN000021	VILLCN000023	VILLAZ000005	VILLCG000005
VILLCN000025	VILLCN000026	VILLCN000027	VILLCN000028	VILLAZ000006	VILLCG000006
VILLCN000030	VILLCN000031	VILCN000032	VILLCN000034	VILLAZ000007	VILLCG000007
VILLCN000035	VILLCN000036	VILLCN000037	VILLCN000038	VILLIR000001	VILLCG000008
VILLCN000039	VILLCN000040	VILLCN000042	VILLCN000044	VILLIR000002	VILLCG000009
VILLCN000046	VILLCN000047	VILLCN000048	VILLCN000049	VILLIR000003	VILLCG000010
VILLCN000050	VILLCN000051	VILLCN000052	VILLCN000053	VILLIR000004	VILLCG000011
VILLCN000054	VILLCN000055	VILLCN000056	VILLCN000057	VILLIR000005	VILLCG000012
VILLCN000058	VILLCN000059	VILLCN000060	VILLCN000062	VILLIR000006	VILLCG000013
VILLCN000063	VILLCN000064	VILLCN000065	VILLCN000066	VILLIR000007	VILLCG000014
VILLCN000068	VILLCN000069	VILLCN000070	VILLCN000071	VILLIR000008	VILLCG000015
VILLCN000072	VILLCN000073	VILLCN000074	VILLCN000075	VILLIR000009	VILLCG000016
VILLCN000078	VILLCN000079	VILLCN000080	VILLCN000081	VILLIR000011	VILLKE000001
VILLCN000084	VILLCN000086	VILLCN000087	VILLCN000088	VILLIR000012	VILLKE000002
VILLCN000089	VILLCN000090	VILLCN000091	VILLCN000092	VILLIR000013	VILLKE000003
VILLCN000093	VILLCN000094	VILLCN000097	VILLCN000099	VILLIR000014	VILLKE000004
VILLCN000100	VILLCN000103	VILLCN000104	VILLCN000108	VILLIR000015	VILLKE000005
VILLCN000109	VILLCN000111	VILLCN000112	VILLCN000114	VILLIR000016	VILLKE000006
VILLCN000115	VILLCN000116	VILLCN000117	VILLCN000118	VILLIR000017	VILLKE000007
VILLCN000120	VILLCN000121	VILLCN000123	VILLCN000124	VILLIR000018	VILLKE000008
VILLCN000125	VILLCN000126	VILLCN000128	VILLCN000129	VILLIR000019	VILLKE000009
VILLCN000130	VILLCN000131	VILLCN000132	VILLCN000133	VILLIR000020	VILLKE000010
VILLCN000134	VILLCN000135	VILLCN000136	VILLCN000137	VILLIR000021	VILLKE000011
VILLCN000139	VILLCN000140	VILLCN000142	VILLCN000143	VILLIR000022	VILLKE000012
VILLCN000144	VILLCN000145	VILLCN000146	VILLCN000147	VILLIR000023	VILLKE000013
VILLCN000148	VILLCN000152	VILLCN000153	VILLCN000154	VILLIR000025	VILLKE000014
VILLKH000001	VILLKH000002	VILLKH000003	VILLKH000004	VILLIR000026	VILLKE000015
VILLMM000004	VILLMM000005	VILLNP000001	VILLNP000002	VILLIR000028	VILLKE000016
VILLNP000003	VILLNP000004	VILLNP000005	VILLNP000006	VILLIR000030	VILLKE000017
VILLNP000007	VILLNP000008	VILLTH00002	VILLTH000005	VILLTJ000001	VILLKE000019
VILLTH000006	VILLTH000008	VILLTH000009		VILLUZ000001	VILLLR000001
				VILLUZ000002	VILLLR000002
				VILLUZ000003	VILLLR000003
				VILLUZ000004	VILLLR000004
				VILLUZ000005	VILLLR000005
				VILLUZ000006	VILLLR000006
					VILLLR000007
					VILLIR000008
					VILLLR000009
					VILLLR000010
					VILLLR000011
					VILLLR000012
					VILLLR000013
					VILLLR000014
					VILLLR000015
					VILLLR000016

Supplementary Table 2. Breed Dog SNP Distances
SNP distances were calculated between one dog randomly selected from each analyzed breed. The minimum, maximum, mean, and median results of the comparisons are shown for each breed.

Breed Name	Minimum	Mean	Median	Maximum
Cocker Spaniel	1,561,755	1,694,320	1,671,977	2,115,342
American Eskimo Dog	1,590,627	1,686,893	1,668,605	2,092,240
Affenpinscher	1,533,830	1,697,714	1,675,342	2,105,508
Afghan Hound	1,723,167	1,837,167	1,825,825	2,141,371
American Foxhound	1,437,571	1,652,102	1,637,385	2,091,209
American Hairless Terrier	1,570,635	1,675,535	1,653,567	2,083,619
Airedale Terrier	1,662,203	1,746,878	1,723,741	2,159,507
Akbash	1,626,080	1,728,518	1,713,258	2,080,192
Japanese Akita	1,438,805	2,024,604	2,026,653	2,227,931
American Akita	1,438,805	1,997,714	1,999,429	2,203,001
Alpine Dachsbracke	1,594,575	1,686,243	1,664,297	2,098,755
Alaskan Malamute	1,512,856	1,918,786	1,915,894	2,138,102
American Bulldog	1,391,027	1,664,364	1,648,888	2,096,842
American Staffordshire Terrier	1,464,659	1,656,107	1,639,106	2,098,154
American Water Spaniel	1,535,998	1,674,361	1,653,804	2,099,800
Anatolian Shepherd Dog	1,644,556	1,766,674	1,752,345	2,102,797
Appenzeller Sennenhund	1,554,004	1,748,469	1,726,667	2,145,059
Artois Hound	1,515,284	1,690,477	1,673,187	2,119,880
Ariegeois	1,430,344	1,626,185	1,606,564	2,073,452
Ariege Pointer	1,483,614	1,669,549	1,650,505	2,102,592
Silky Terrier	1,536,733	1,681,655	1,659,247	2,101,459
Australian Cattle Dog	1,606,202	1,697,732	1,676,907	2,101,109
Auvergne Pointer	1,535,668	1,654,623	1,633,111	2,090,715
Australian Kelpie	1,575,297	1,669,692	1,647,102	2,092,434
Australian Shepherd	1,575,895	1,680,296	1,657,620	2,088,079
Australian Terrier	1,538,012	1,685,469	1,664,701	2,091,833
Basset Artesien Normand	1,438,154	1,697,190	1,677,850	2,118,607
Basset Hound	1,438,154	1,683,399	1,664,963	2,101,859
White Swiss Shepherd Dog	1,162,708	1,682,604	1,672,702	2,101,087
Beauceron	1,581,119	1,670,400	1,648,748	2,106,902
Belgian Tervuren	1,378,416	1,664,012	1,644,161	2,081,946
Bedlington Terrier	1,630,948	1,723,028	1,699,071	2,136,263
Belgian Sheepdog	1,100,241	1,694,446	1,674,037	2,107,270
Bergamasco Sheepdog	1,560,095	1,651,025	1,630,440	2,082,451
Basset Fauve de Bretagne	1,466,045	1,662,397	1,642,150	2,092,049
Blue Gascony Griffon	1,430,344	1,621,259	1,604,908	2,065,223
Bohemian Shepherd	1,189,208	1,729,101	1,719,125	2,132,483
Bichon Frise	1,580,008	1,688,462	1,667,066	2,103,585
Havanese	1,495,989	1,656,045	1,636,221	2,075,068
Billy	1,195,380	1,659,373	1,646,568	2,085,884
Bloodhound	1,509,127	1,691,883	1,674,750	2,118,099
Russian Tsvetnaya Bolonka	1,613,633	1,695,358	1,677,869	2,073,532
Blue Picardy Spaniel	1,279,443	1,659,718	1,640,192	2,090,311

**Bluetick Coonhound**	1,480,782	1,648,809	1,628,257	2,085,776
**Belgian Malinois**	1,418,225	1,655,842	1,637,280	2,086,034
**Bavarian Mountain Scent Hound**	1,542,663	1,672,418	1,650,953	2,096,019
**Bouvier des Ardennes**	1,508,874	1,657,091	1,637,853	2,075,391
**Boerboel**	1,417,226	1,646,636	1,626,805	2,086,394
**Bolognese**	1,545,256	1,657,826	1,638,938	2,080,650
**Bourbonnais Pointing Dog**	1,502,749	1,673,108	1,653,634	2,113,155
**Boston Terrier**	1,473,671	1,659,711	1,641,234	2,098,397
**Bouvier des Flandres**	1,582,781	1,689,535	1,665,215	2,109,776
**Boxer**	1,406,464	1,719,292	1,704,845	2,145,332
**Boykin Spaniel**	1,535,998	1,674,030	1,651,449	2,099,669
**Bracco Italiano**	1,483,614	1,685,613	1,667,189	2,104,935
**Barbet**	1,561,067	1,655,433	1,632,890	2,090,879
**Braques Francais**	1,443,750	1,640,969	1,620,523	2,082,922
**Briquet Griffon Vendeen**	1,318,368	1,652,911	1,636,843	2,089,797
**Broholmer**	1,554,128	1,693,555	1,670,083	2,114,599
**Briard**	1,581,119	1,688,851	1,666,237	2,112,870
**Brittany**	1,491,890	1,661,835	1,642,919	2,087,104
**Bruno Jura Hound**	1,458,197	1,653,931	1,633,249	2,092,756
**Bernese Mountain Dog**	1,547,768	1,693,370	1,668,947	2,101,308
**Black Russian Terrier**	1,522,173	1,668,695	1,647,280	2,086,298
**Brussels Griffon**	1,115,238	1,685,894	1,664,499	2,099,124
**Brazilian Terrier**	1,546,312	1,667,549	1,645,103	2,099,597
**Black and Tan Coonhound**	1,536,950	1,683,232	1,663,581	2,104,593
**French Bulldog**	1,453,522	1,675,719	1,658,800	2,109,543
**English Bulldog**	1,037,054	1,690,575	1,679,717	2,121,233
**Bullmastiff**	1,404,997	1,696,800	1,678,507	2,122,647
**Bull Terrier**	785,014	1,732,413	1,718,354	2,154,921
**Biewer Terrier**	1,370,539	1,678,674	1,655,855	2,106,140
**Canaan Dog**	1,672,740	1,765,371	1,750,843	2,111,670
**Cairn Terrier**	1,588,516	1,683,524	1,661,465	2,103,382
**Dogo Canario**	1,455,013	1,649,083	1,630,599	2,092,889
**Cardigan Welsh Corgi**	1,525,609	1,667,733	1,646,342	2,090,284
**Schipperke**	1,625,671	1,706,206	1,685,230	2,100,429
**Central Asian Shepherd Dog**	1,689,965	1,778,310	1,766,056	2,080,962
**Caucasian Ovcharka**	1,626,508	1,721,673	1,706,864	2,079,431
**Curly-Coated Retriever**	1,603,604	1,693,264	1,669,355	2,105,630
**Cesky Fousek**	1,423,240	1,643,719	1,623,610	2,088,007
**Chesapeake Bay Retriever**	1,545,676	1,645,368	1,623,026	2,073,199
**Chihuahua**	1,570,526	1,668,122	1,649,101	2,076,583
**Japanese Chin**	1,742,654	1,811,800	1,800,000	2,071,005
**Chow Chow**	1,803,537	2,051,457	2,054,853	2,241,064
**Cirneco dell'Etna**	1,511,162	1,687,299	1,666,587	2,101,063
**Cavalier King Charles Spaniel**	1,241,321	1,710,505	1,689,468	2,131,413
**Clumber Spaniel**	1,626,628	1,732,527	1,709,892	2,149,045
**Cane Corso**	1,434,395	1,653,388	1,635,616	2,081,034
**Continental Bulldog**	1,037,054	1,668,280	1,657,551	2,105,760

**Collie**	1,305,666	1,731,757	1,712,324	2,135,479
**Chinook**	1,524,614	1,765,515	1,751,152	2,117,645
**Coton de Tulear**	1,586,772	1,672,677	1,651,347	2,075,975
**Chinese Crested**	1,582,238	1,674,129	1,654,009	2,085,003
**Croatian Sheepdog**	1,569,870	1,659,602	1,639,508	2,075,685
**Cretan Tracer**	1,574,775	1,675,836	1,657,390	2,082,306
**Hellenic Hound**	1,563,836	1,664,244	1,646,449	2,073,639
**Catalan Sheepdog**	1,592,756	1,675,202	1,653,712	2,088,476
**Cesky Terrier**	1,532,756	1,736,933	1,714,774	2,141,374
**Catalburun**	1,534,037	1,640,893	1,623,079	2,070,533
**Czechoslovakian Wolfdog**	1,565,861	1,962,599	1,960,198	2,286,807
**Dachshund**	1,573,066	1,663,161	1,641,739	2,086,515
**Dalmatian**	1,582,955	1,675,805	1,653,283	2,098,612
**Dandie Dinmont Terrier**	1,630,948	1,710,712	1,688,315	2,121,039
**Dogo Argentino**	1,539,827	1,685,106	1,665,854	2,093,115
**Dogue de Bordeaux**	1,519,635	1,693,230	1,675,919	2,108,798
**Scottish Deerhound**	1,588,522	1,717,737	1,694,975	2,136,517
**Doberman Pinscher**	1,644,450	1,722,249	1,701,225	2,121,027
**Drever**	1,589,861	1,685,101	1,663,113	2,095,039
**Danish-Swedish Farmdog**	1,514,772	1,636,866	1,614,851	2,075,159
**Dutch Shepherd**	1,505,805	1,681,959	1,662,086	2,093,386
**Dutch Partridge Dog**	1,587,443	1,697,549	1,675,434	2,114,743
**English Cocker Spaniel**	1,465,573	1,688,588	1,665,828	2,117,624
**East-European shepherd**	1,113,194	1,670,329	1,662,429	2,092,763
**English Foxhound**	1,291,903	1,676,730	1,665,398	2,111,897
**English Toy Spaniel**	1,241,321	1,692,779	1,671,288	2,109,739
**English Shepherd**	1,553,894	1,655,736	1,634,752	2,075,702
**English Toy Terrier**	1,444,568	1,670,315	1,647,968	2,103,438
**Elo**	1,587,233	1,744,434	1,734,779	2,004,826
**Entlebucher Mountain Dog**	1,554,004	1,741,456	1,719,467	2,140,338
**English Pointer**	1,510,478	1,690,356	1,671,281	2,125,552
**English Setter**	1,479,729	1,687,559	1,667,456	2,118,395
**Estrela Mountain Dog**	1,568,368	1,660,610	1,640,543	2,075,570
**English Springer Spaniel**	1,590,399	1,686,545	1,663,413	2,105,343
**Estonian Hound**	1,547,273	1,670,729	1,650,581	2,089,888
**Eurasier**	1,587,233	1,845,376	1,838,829	2,055,724
**Field Spaniel**	1,465,573	1,702,796	1,680,451	2,128,243
**Finnish Hound**	1,553,232	1,678,386	1,659,350	2,087,701
**Finnish Spitz**	1,622,738	1,758,236	1,743,467	2,088,917
**Flat-Coated Retriever**	1,566,636	1,710,036	1,686,133	2,123,911
**Formosan Mountain Dog**	1,781,560	1,856,148	1,849,386	2,063,321
**French Spaniel**	1,567,236	1,676,550	1,653,750	2,108,038
**Wire Fox Terrier**	1,269,032	1,708,310	1,690,091	2,121,978
**Smooth Fox Terrier**	1,452,735	1,703,677	1,684,018	2,119,560
**Great Anglo-French Tricolour Hound**	1,229,958	1,673,511	1,660,171	2,097,550
**Galgo Espanol**	1,522,177	1,649,698	1,629,102	2,073,594
**Great Anglo-French White and Orange Hound**	1,195,380	1,643,994	1,633,441	2,087,306

**Grand Bleu de Gascogne**	1,413,871	1,654,487	1,636,958	2,096,851
**Grand Basset Griffon Vendeen**	1,318,161	1,681,808	1,661,637	2,108,880
**Griffon Fauve de Bretagne**	1,466,045	1,636,848	1,616,745	2,075,233
**Grand Griffon Vendeen**	1,318,368	1,665,845	1,651,719	2,098,685
**German Hound**	1,603,460	1,701,587	1,678,520	2,116,864
**German Hunting Terrier**	1,465,455	1,661,826	1,642,639	2,092,039
**Glen of Imaal Terrier**	1,543,988	1,687,440	1,666,739	2,111,919
**German Spaniel**	1,601,168	1,689,753	1,668,080	2,104,459
**Golden Retriever**	1,575,976	1,678,466	1,655,233	2,097,228
**Gordon Setter**	1,504,571	1,653,029	1,632,959	2,090,477
**German Pinscher**	1,502,220	1,680,592	1,658,244	2,102,027
**Great Pyrenees**	1,527,484	1,706,198	1,686,358	2,111,346
**Greenland Dog**	1,512,856	1,959,934	1,958,030	2,160,796
**Groenendael**	1,100,241	1,703,658	1,684,623	2,110,020
**Griffon Nivernais**	1,469,782	1,635,673	1,618,415	2,070,582
**German Shepherd Dog**	1,113,194	1,674,329	1,666,857	2,097,873
**German Shorthaired Pointer**	1,443,750	1,650,253	1,631,075	2,098,229
**Greater Swiss Mountain Dog**	1,547,768	1,728,133	1,707,022	2,138,509
**Giant Schnauzer**	1,522,173	1,669,596	1,647,075	2,086,089
**German Spitz Klein**	1,423,291	1,659,550	1,640,558	2,065,213
**German Spitz**	1,413,425	1,678,686	1,660,328	2,062,964
**German Giant Spitz**	1,413,425	1,669,797	1,651,373	2,069,294
**Pomeranian**	1,423,291	1,665,841	1,647,144	2,078,092
**German Spitz Mittel**	1,451,413	1,672,110	1,652,866	2,073,014
**Gotland Hound**	1,378,156	1,672,947	1,655,231	2,097,996
**German Wirehaired Pointer**	1,423,240	1,660,779	1,642,318	2,098,078
**Hallefors Elkhound**	1,530,119	1,761,156	1,745,778	2,087,859
**Harrier**	1,291,903	1,652,683	1,640,224	2,097,566
**Hortaya Borzaya**	1,494,745	1,702,707	1,685,484	2,085,801
**Hokkaido**	1,866,895	2,023,324	2,024,277	2,223,790
**Hovawart**	1,524,408	1,692,795	1,673,056	2,110,069
**Vizsla**	1,539,721	1,662,008	1,641,340	2,088,355
**Hamiltonstovare**	1,378,156	1,672,445	1,656,047	2,093,894
**Hanoverian Scenthound**	1,542,663	1,705,383	1,683,132	2,111,093
**Ibizan Hound**	1,571,542	1,691,266	1,672,811	2,100,482
**Icelandic Sheepdog**	1,631,391	1,712,098	1,695,364	2,082,349
**Irish Terrier**	1,613,690	1,720,633	1,698,241	2,132,831
**Irish Setter**	1,425,994	1,657,644	1,636,837	2,094,200
**Irish Water Spaniel**	1,585,794	1,687,830	1,662,828	2,121,197
**Japanese Spitz**	1,608,360	1,721,509	1,703,278	2,090,128
**Kai Ken**	1,816,170	1,884,044	1,881,268	2,103,943
**Kangal**	1,625,814	1,736,301	1,723,035	2,086,815
**Kars**	1,687,702	1,772,245	1,757,525	2,100,607
**Keeshond**	1,572,698	1,678,971	1,659,869	2,088,814
**Kerry Blue Terrier**	1,556,894	1,667,452	1,644,594	2,097,249
**Kintamani**	1,727,275	2,068,325	2,072,517	2,249,877
**Kishu**	1,829,849	1,992,600	1,992,156	2,193,852

**Small Munsterlander**	1,532,663	1,660,068	1,637,077	2,088,198
**Kromfohrlander**	1,620,825	1,707,955	1,686,108	2,118,113
**Kuvasz**	1,607,060	1,688,113	1,669,445	2,088,193
**Labrador Retriever**	1,566,636	1,671,412	1,646,749	2,086,764
**Belgian Laekenois**	1,527,649	1,691,050	1,671,063	2,095,821
**Lagotto Romagnolo**	1,559,425	1,655,095	1,633,276	2,076,306
**Lakeland Terrier**	1,269,032	1,707,015	1,687,829	2,125,381
**Lancashire Heeler**	1,559,343	1,676,378	1,654,672	2,092,293
**Landseer**	1,423,144	1,674,803	1,653,985	2,091,396
**Leonberger**	1,576,641	1,676,365	1,655,116	2,090,782
**Catahoula Leopard Dog**	1,525,611	1,639,220	1,618,351	2,077,539
**Lhasa Apso**	1,585,567	1,784,035	1,775,410	2,045,150
**Large Munsterlander**	1,532,663	1,665,304	1,645,623	2,082,536
**Lowchen**	1,599,204	1,691,272	1,669,881	2,104,864
**Magyar Agar**	1,522,177	1,665,023	1,643,218	2,090,439
**Maltese**	1,495,989	1,699,611	1,679,408	2,098,995
**Manchester Terrier (Standard)**	1,444,568	1,718,598	1,696,591	2,132,500
**Maremma Sheepdog**	1,582,656	1,676,406	1,656,712	2,088,890
**Mastiff**	1,404,997	1,686,695	1,668,807	2,113,316
**Miniature Bull Terrier**	785,014	1,722,512	1,708,715	2,145,531
**Mountain Cur**	1,477,764	1,619,579	1,597,949	2,044,039
**Majorca Mastiff**	1,296,896	1,665,191	1,647,296	2,094,830
**Miniature Pinscher**	1,511,804	1,662,179	1,640,443	2,093,378
**Miniature Poodle**	1,278,200	1,650,741	1,631,300	2,079,233
**Miniature Schnauzer**	1,635,407	1,721,309	1,699,108	2,124,985
**Mudi**	1,592,332	1,675,798	1,656,794	2,092,075
**Norwegian Buhund**	1,635,933	1,715,257	1,694,997	2,088,170
**Neapolitan Mastiff**	1,441,461	1,694,136	1,672,394	2,112,599
**Norwegian Elkhound**	1,530,119	1,733,323	1,715,718	2,103,765
**Newfoundland**	1,423,144	1,679,422	1,656,727	2,091,879
**Norwegian Lundehund**	1,818,217	1,863,881	1,841,361	2,235,905
**Norrbottenspitz**	1,601,151	1,697,974	1,680,758	2,065,709
**Norfolk Terrier**	1,393,679	1,745,182	1,723,342	2,151,228
**Norwich Terrier**	1,393,679	1,712,696	1,691,164	2,131,242
**Nova Scotia Duck Tolling Retriever**	1,563,289	1,671,223	1,647,518	2,085,208
**Olde English Bulldogge**	1,215,573	1,692,082	1,679,381	2,124,896
**Old English Sheepdog**	1,583,402	1,677,102	1,654,690	2,093,486
**Old German Shepherd**	1,381,222	1,648,249	1,632,639	2,065,642
**Otterhound**	1,448,203	1,705,021	1,690,389	2,120,067
**Continental Toy Spaniel (Papillon)**	1,307,814	1,658,100	1,637,684	2,081,488
**Parson Russell Terrier**	1,457,895	1,624,551	1,603,084	2,066,260
**Pont-Audemer spaniel**	1,421,729	1,648,798	1,627,619	2,081,478
**Patterdale Terrier**	1,543,034	1,683,189	1,660,651	2,103,058
**Petit Bleu de Gascogne**	1,413,871	1,643,205	1,624,886	2,078,415
**Brussels Griffon (smooth)**	1,115,238	1,681,683	1,660,923	2,098,573
**Petit Basset Griffon Vendeen**	1,318,161	1,667,989	1,650,090	2,099,949
**Pekingese**	1,572,450	1,775,389	1,764,020	2,066,654

**Pembroke Welsh Corgi**	1,525,609	1,703,775	1,680,511	2,110,034
**Peruvian Hairless**	1,562,874	1,651,664	1,630,959	2,069,463
**Continental Toy Spaniel (Phalene)**	1,307,814	1,664,073	1,641,792	2,079,370
**Pharaoh Hound**	1,511,162	1,709,484	1,689,789	2,117,373
**Picardy Shepherd**	1,511,083	1,729,170	1,711,933	2,141,394
**Picardy Spaniel**	1,279,443	1,661,839	1,642,867	2,102,366
**Plott Hound**	1,480,686	1,636,366	1,616,749	2,075,106
**Polish Greyhound**	1,494,745	1,684,614	1,667,422	2,087,993
**Polish Lowland Sheepdog**	1,599,008	1,695,675	1,675,118	2,096,807
**Polish Tatra Sheepdog**	1,591,328	1,685,690	1,665,023	2,087,732
**Poodle**	1,278,200	1,651,487	1,632,047	2,070,663
**Portuguese Pointer**	1,539,140	1,673,605	1,654,056	2,091,380
**Porcelaine**	1,557,543	1,700,997	1,683,246	2,121,035
**Portuguese Sheepdog**	1,584,479	1,667,301	1,646,863	2,088,894
**Posavac Hound**	1,542,432	1,652,359	1,633,425	2,071,286
**Portuguese Podengo**	1,581,757	1,676,794	1,657,169	2,082,879
**Portuguese Podengo Pequeno**	1,556,928	1,652,278	1,630,863	2,081,152
**Prague Ratter**	1,502,220	1,670,970	1,650,772	2,081,456
**Portuguese Water Dog**	1,579,561	1,666,218	1,644,617	2,088,644
**Pudelpointer**	1,520,496	1,660,412	1,640,347	2,085,674
**Pug**	1,624,783	1,736,267	1,716,245	2,130,195
**Puli**	1,581,448	1,669,799	1,649,296	2,074,083
**Pumi**	1,573,416	1,665,255	1,647,025	2,074,494
**Pyrenean Mastiff**	1,527,484	1,670,683	1,652,226	2,081,918
**Pyrenean Shepherd**	1,599,617	1,681,198	1,660,091	2,093,618
**Ratonero Bodeguero Andaluz**	1,542,692	1,649,237	1,628,478	2,079,163
**Rat Terrier**	1,518,762	1,655,765	1,635,037	2,075,810
**Redbone Coonhound**	1,478,193	1,636,826	1,617,814	2,081,288
**Rhodesian Ridgeback**	1,584,309	1,678,630	1,655,422	2,095,075
**Teddy Roosevelt Terrier**	1,504,510	1,628,367	1,608,228	2,058,300
**Russian Toy**	1,560,909	1,655,854	1,636,595	2,075,731
**Russian Hound**	1,575,133	1,691,753	1,673,687	2,086,874
**Irish Red and White Setter**	1,425,994	1,674,764	1,653,807	2,106,237
**Saarloos Wolfdog**	1,675,219	1,966,339	1,959,437	2,262,979
**Saluki**	1,655,336	1,794,958	1,781,198	2,123,990
**Samoyed**	1,729,423	1,816,479	1,806,191	2,085,308
**Sarplaninac**	1,612,233	1,703,329	1,687,463	2,082,315
**Schapendoes**	1,602,070	1,689,973	1,667,240	2,107,102
**Sealyham Terrier**	1,532,756	1,701,094	1,679,059	2,115,029
**Segugio Italiano**	1,521,347	1,647,400	1,626,909	2,083,792
**Chinese Shar-Pei**	1,803,596	1,975,712	1,975,357	2,167,771
**Shiba Inu**	1,816,559	1,980,849	1,980,924	2,193,241
**Shih Tzu**	1,572,450	1,756,460	1,745,166	2,046,157
**Shiloh Shepherd**	1,163,733	1,713,838	1,705,384	2,121,896
**Shikoku**	1,842,164	2,090,039	2,092,595	2,286,807
**Skye Terrier**	1,648,946	1,726,252	1,702,262	2,134,834
**Silken Windhound**	1,494,490	1,692,574	1,671,881	2,105,851

**Swedish Lapphund**	1,626,936	1,716,146	1,696,914	2,085,392
**Slovak Cuvac**	1,599,025	1,694,080	1,674,826	2,097,183
**Slovensky Kopov**	1,597,496	1,682,945	1,660,981	2,100,998
**Sloughi**	1,574,476	1,690,072	1,672,591	2,081,070
**Slovakian Wirehaired Pointer**	1,458,887	1,666,708	1,646,282	2,091,535
**Saint Miguel Cattle Dog**	1,558,803	1,656,341	1,635,691	2,069,036
**Small Swiss Hound**	1,559,471	1,699,561	1,677,876	2,114,360
**Spinone Italiano**	1,552,600	1,652,200	1,632,408	2,079,181
**Spanish Mastiff**	1,570,948	1,666,401	1,647,101	2,080,353
**Spanish Water Dog**	1,557,400	1,657,217	1,637,999	2,075,647
**Sarabi**	1,677,412	1,766,890	1,752,533	2,106,539
**South Russian Ovcharka**	1,619,668	1,712,152	1,694,139	2,103,369
**Shetland Sheepdog**	1,305,666	1,698,456	1,678,344	2,121,209
**Standard Schnauzer**	1,597,505	1,683,567	1,662,397	2,087,853
**Staffordshire Bull Terrier**	1,484,020	1,676,571	1,659,894	2,110,075
**Saint Bernard**	1,576,641	1,681,397	1,659,276	2,093,308
**Stabyhoun**	1,627,928	1,707,620	1,683,432	2,118,738
**Saint-Usuge Spaniel**	1,421,729	1,641,834	1,621,149	2,080,620
**Sussex Spaniel**	1,632,441	1,748,102	1,723,465	2,163,092
**Swedish Vallhund**	1,636,875	1,718,077	1,697,261	2,108,122
**Swedish Elkhound**	1,512,759	1,699,257	1,679,023	2,089,519
**Swiss Hound**	1,458,197	1,670,230	1,650,481	2,091,526
**Swedish White Elkhound**	1,512,759	1,722,971	1,703,944	2,096,412
**Tazi**	1,655,336	1,769,507	1,756,808	2,103,636
**Thai Ridgeback**	1,727,275	2,000,901	1,999,847	2,203,478
**Tibetan Spaniel**	1,670,339	1,835,461	1,827,310	2,085,835
**Tibetan Terrier**	1,663,099	1,747,748	1,732,245	2,072,655
**Tamaskan**	1,478,816	1,759,795	1,752,611	2,057,238
**Tosa Inu**	1,594,706	1,724,364	1,711,283	2,009,837
**Toy Poodle**	1,344,958	1,646,908	1,627,052	2,070,613
**Turkish Mastiff**	1,625,814	1,734,701	1,719,021	2,091,816
**Treeing Walker Coonhound**	1,475,916	1,635,643	1,616,182	2,082,492
**Toy Fox Terrier**	1,513,563	1,648,017	1,624,411	2,085,730
**Volpino Italiano**	1,554,115	1,699,126	1,680,358	2,090,745
**Weimaraner**	1,458,887	1,693,488	1,671,444	2,110,554
**Welsh Terrier**	1,317,199	1,679,356	1,659,270	2,103,829
**Whippet**	1,494,490	1,681,951	1,658,850	2,113,472
**Wirehaired Pointing Griffon**	1,541,944	1,671,725	1,651,537	2,106,995
**Welsh Springer Spaniel**	1,580,804	1,693,527	1,670,992	2,112,770
**Xoloitzcuintli**	1,534,222	1,630,876	1,612,681	2,059,468
**Yorkshire Terrier**	1,370,539	1,669,889	1,649,305	2,091,414
**Zagar**	1,558,818	1,666,211	1,647,602	2,075,948
**Turkish Zerdava**	1,624,104	1,716,636	1,701,487	2,084,392

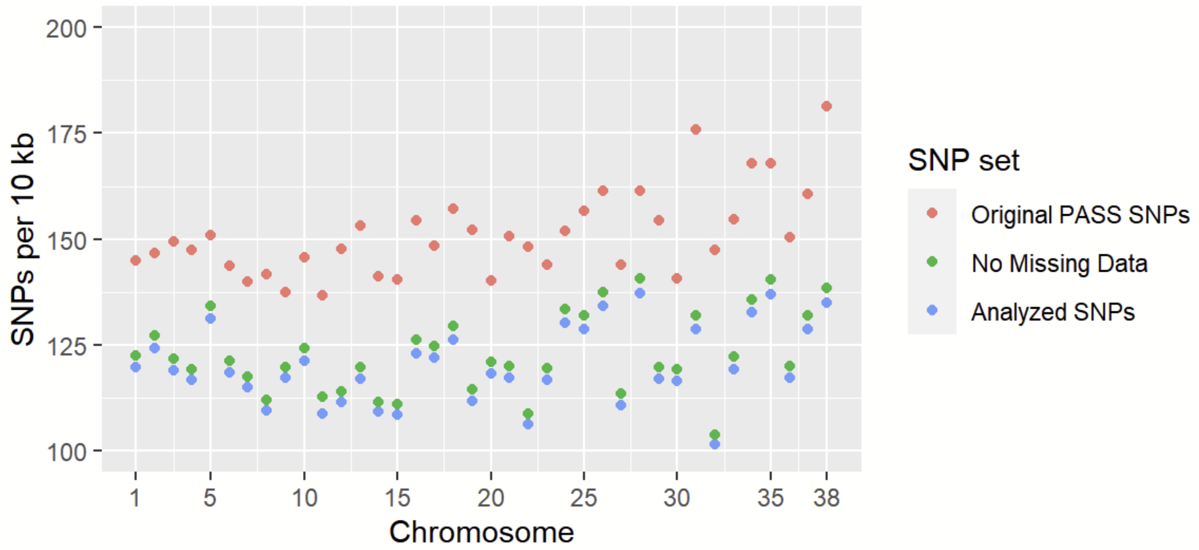

Figure S1. Density of SNPs included in canine analysis
The density of SNPs per 10 kb is shown for different filtration sets across the 38 canine autosomes. The red points represent all sites listed as ‘PASS’ in the initial VCF file. The green points represent sites with no missing data in the 1,929 considered samples. The blue points represent the final variant set used for downstream analysis.

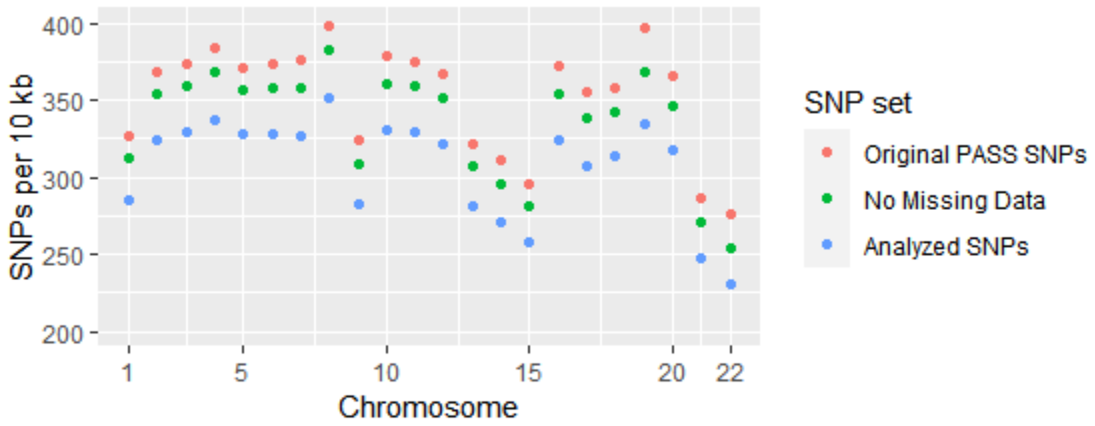

Figure S2. Density of SNPs from the Human 1000 Genomes Project
The rate of original PASS SNPs, SNPs with no missing data, and analyzed SNPs, from the high-coverage 1000 Genomes Project data set is shown.

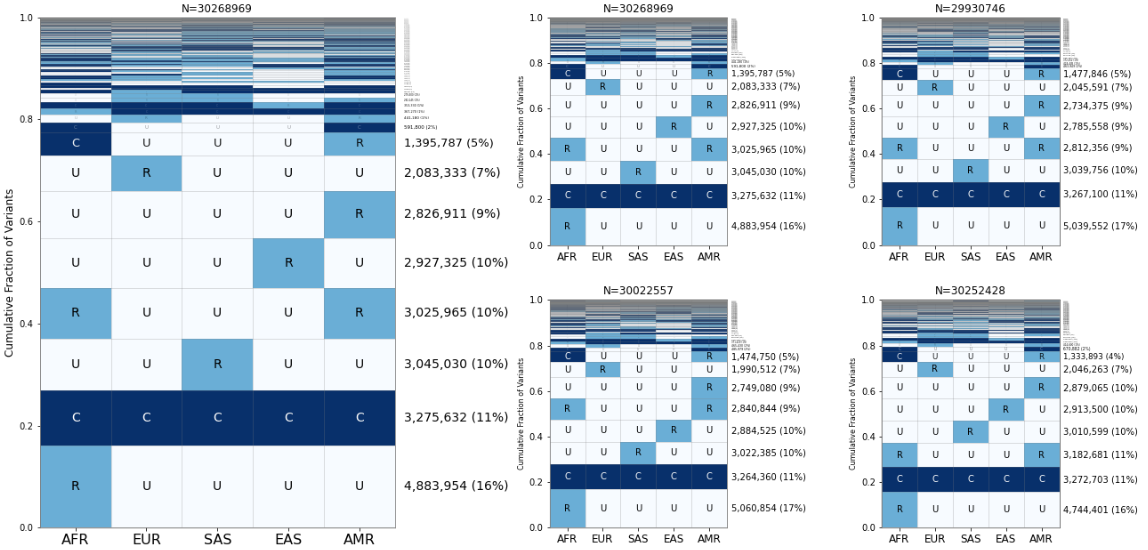

Figure S3. Human GeoVar analysis with reduced sample sizes

A similar analysis to that of Figure 3 but with the human 1000 Genomes Project data, arranged in the same five populations as Biddanda *et. al*. Population codes: AFR=Africa, EUR=Europe, SAS=South Asia, EAS=East Asia, AMR=Americas. A random selection of 50 individuals per group was analyzed.

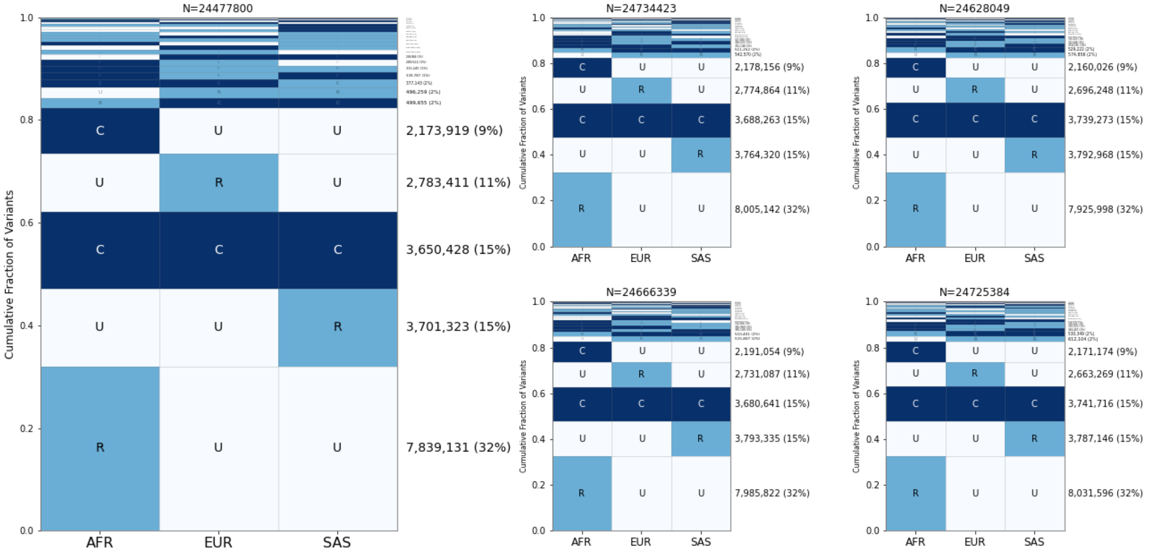

Figure S4. Human GeoVar analysis with three groups

The same analysis as Figure S2, but with three populations instead of five. The South and East Asian populations have been combined into 'SAS', and the America population has been removed.

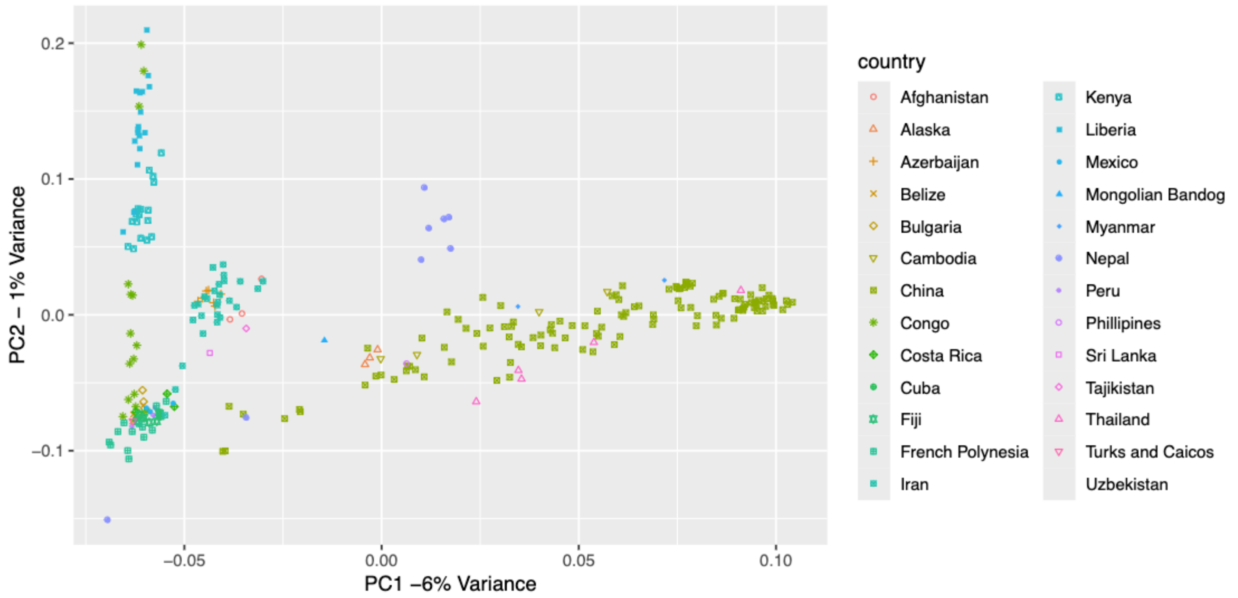

Figure S5. Principal component analysis of village dogs

The three groups analyzed as populations are shown with the points colored based on sample location. Apart from the Chinese village dogs, which show a wide range of PC1 values, and were categorized according to the grouping shown, all village dogs from one country were categorized into one of the three categories, or not categorized at all. The categories are: Africa = Congo, Liberia, Kenya; Central Asia = Afghanistan, Azerbaijan, Bulgaria, Iran, Tajikistan, Uzbekistan, East Asia = Cambodia, China (most samples), Myanmar, Nepal, Philippines, Sri Lanka, Thailand. The exact samples in each category are listed in Table S1.
